# Iatrogenic Bronchopleural Fistula

**DOI:** 10.7759/cureus.12187

**Published:** 2020-12-20

**Authors:** Pedro Marques, Gisela Andrade, Joana Granadas, Pedro João, Natacha Abreu

**Affiliations:** 1 Department of Radiology, Hospital Professor Doutor Fernando Fonseca, Amadora, PRT

**Keywords:** bronchopleural fistula, iatrogeny, radiology, chest ct

## Abstract

A bronchopleural fistula (BPF) is a communication between the pleural space and the bronchial tree or the lung parenchyma. Despite being a rare entity, a BPF may carry a high mortality rate. Symptoms of BPF are often nonspecific and subtle, so a high index of clinical suspicion is essential for its correct diagnosis, with imaging playing an extremely important role both in the diagnosis and in the selection of the most appropriate therapeutic approach for each patient. This paper reports a case of a 60-year-old male admitted to the hospital for an etiological investigation of a unilateral pleural effusion. The patient underwent several procedures, among them a video-assisted thoracic surgery, complicated by a peripheral BPF. Therapeutic approach for BPFs must be adapted to each particular case. In this patient, a conservative approach proved to be effective. Meanwhile, the patient was diagnosed with pleural tuberculosis, being discharged on antibacillary medication and while improving BPF’s manifestations.

## Introduction

A bronchopleural fistula (BPF) is a communication between the pleural space and the bronchial tree or the lung parenchyma, and it is a relatively rare pathology. BPF formation encompasses several causes, the most common one is due to complications of lung surgery. BPF may carry high morbidity and mortality rates, with imaging playing an essential role in its diagnosis. This case report presents a 60-year-old male who developed a peripheral BPF after undergoing video-assisted pleural biopsies.

## Case presentation

A 60-year-old male was referred to our institution to study a new left pleural effusion. His medical history revealed active smoking habits (50 pack-year), dyslipidemia, and ischemic heart disease with heart failure. Relevant etiologic investigations done previously consisted of the following: thoracentesis - exudate of lymphocytic predominance, normal adenosine deaminase (ADA) level, irrelevant immunophenotyping changes, negative microbiology, negative cytology, and negative polymerase chain reaction (PCR) for *Mycobacterium tuberculosis*; chest computed tomography (CT) - large left pleural effusion associated with homolateral diffuse homogeneous pleural thickening, without any lung parenchyma neither mediastinal findings; bronchofibroscopy - no endobronchial changes; negative bronchoalveolar lavage microbiological analysis for *M. tuberculosis* (either direct microscopic examination or PCR); and positron emission tomography-CT (PET-CT) - large left pleural effusion with mild metabolic expression, and detection of a small nodularity in the lateral visceral pleura of the left lung with more intense metabolism. Thus, the possibility of a left pleural mesothelioma could not be ruled out.

Given the absence of a definite diagnosis and considering the hypothesis of mesothelioma, the patient was referred to our institution for a pleural biopsy by video-assisted thoracic surgery (VATS). On admission, the patient was clinically stable, with no signs of respiratory distress nor dyspnea. During VATS, 500 milliliters of serofibrinous fluid were aspirated (for biochemistry, microbiology, and cytology tests). The pleural cavity had numerous fibrin adhesions, diffuse thickening of the costal and diaphragmatic pleura, whitish and non-nodular in appearance, and thickening of the visceral pleura as well. Debridement of the entire basal area of the pleural cavity was done. Multiple pleural biopsies (for histological and microbiology testing, namely *Mycobacterium*) were performed and a chest tube was placed in an apical position. Direct bacterial (including acid-alcohol-resistant bacilli) and mycological microscopic examinations of pleural biopsies and pleural fluid were negative.

**Figure 1:**
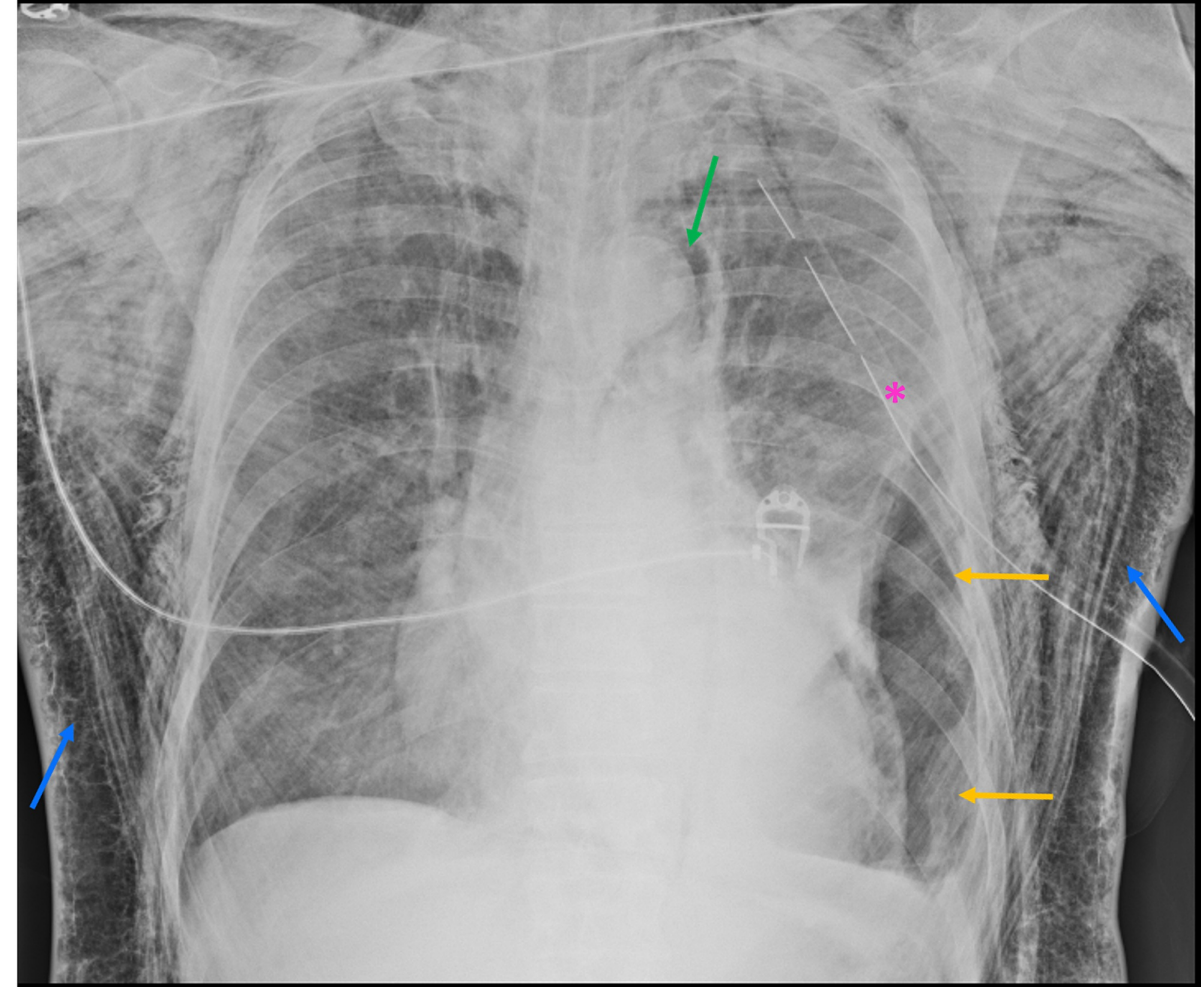
Supine chest radiograph Supine chest radiograph that shows a left basal pneumothorax (yellow arrows), pneumomediastinum (green arrow) and extensive thoracic subcutaneous emphysema (blue arrows). Chest tube (pink asterisk).

About 24 hours post-VATS, the patient developed exuberant subcutaneous emphysema, without other relevant symptoms, namely dyspnea, and remained hemodynamically stable. A chest radiograph was performed, showing left basal pneumothorax and subcutaneous thoracic and cervical emphysema (Figure 1)

**Figure 2:**
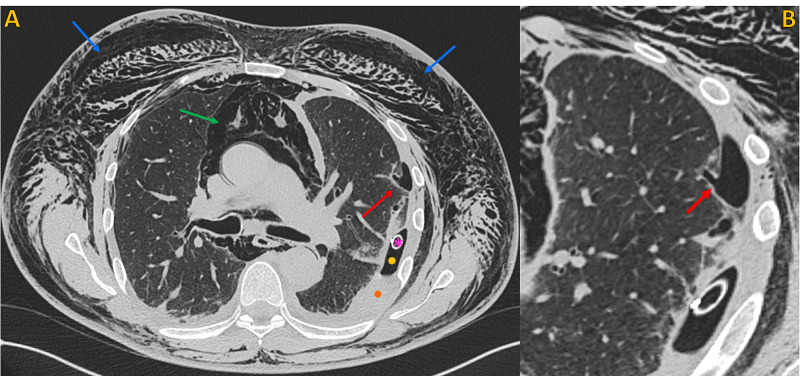
Non-contrast chest CT, lung window Non-contrast chest CT, lung window. A) Axial plane and B) Oblique axial plane (magnified view), demonstrates the point of communication between a peripheral enlarged bronchiolar structure and the pleural space, compatible with a bronchopleural fistula (red arrow). Note extensive soft tissue emphysema (blue arrows), large pneumomediastinum (green arrow), left pneumothorax (yellow circle), and left pleural effusion (orange circle). Chest tube (pink asterisk).

**Figure 3:**
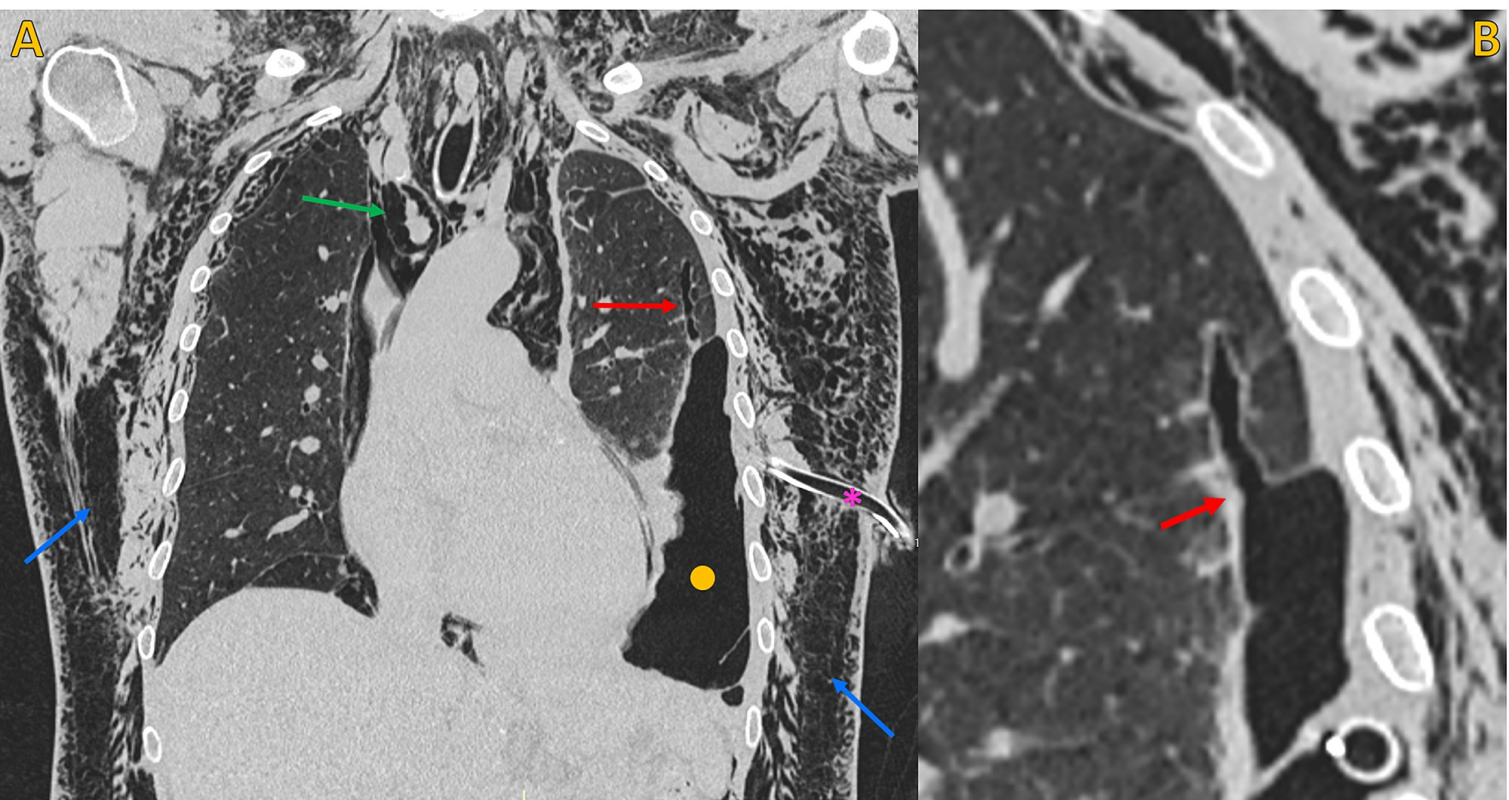
Non-contrast chest CT, lung window Non-contrast chest CT, lung window. A) Coronal plane and B) Oblique coronal plane (magnified view), shows the point of communication between a peripheral enlarged bronchiolar structure and the pleural space, compatible with a bronchopleural fistula (red arrow). Note extensive soft tissue emphysema (blue arrows), large pneumomediastinum (green arrow) and left pneumothorax (yellow circle). Chest tube (pink asterisk).

**Figure 4:**
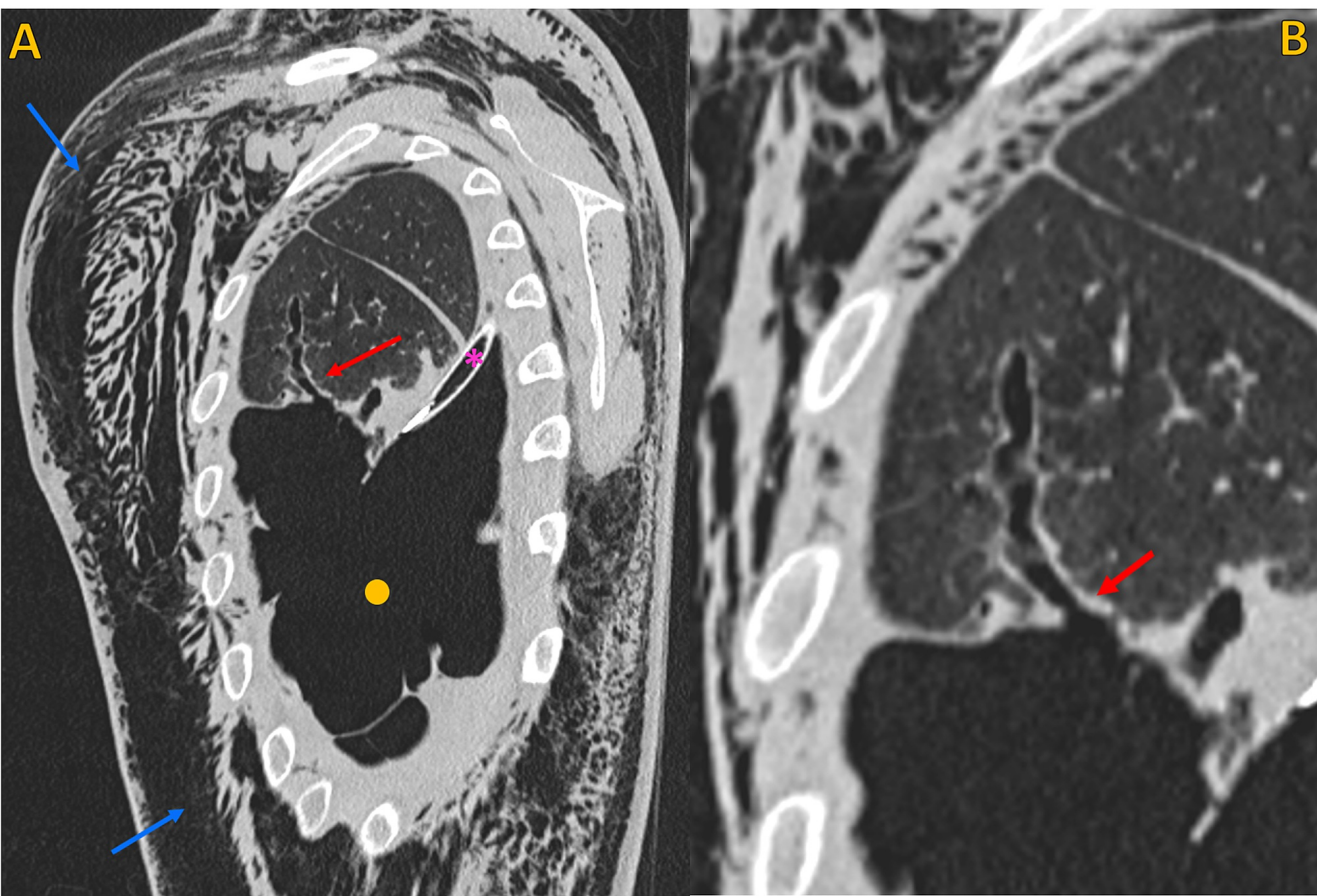
Non-contrast chest CT, lung window Non-contrast chest CT, lung window. A) Oblique sagittal plane and B) Oblique sagittal plane (magnified view), demonstrates the point of communication between a peripheral enlarged bronchiolar structure and the pleural space, compatible with a bronchopleural fistula (red arrow). Note extensive soft tissue emphysema (blue arrows) and left pneumothorax (yellow circle). Chest tube (pink asterisk).

**Figure 5:**
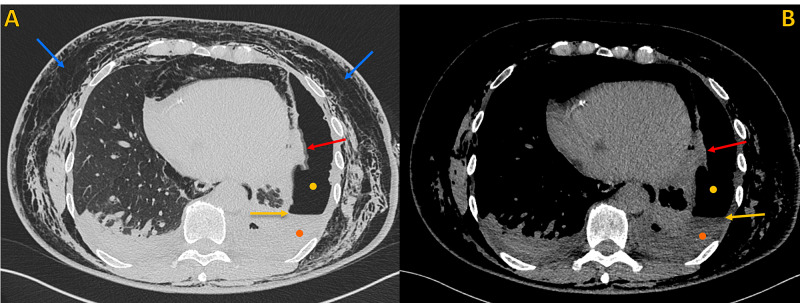
Non-contrast chest CT, same axial plane Non-contrast chest CT, axial images. A) Lung window and B) Soft tissue window, shows focal thickening of the visceral pleura (red arrow) and the air-fluid level of the hydropneumothorax (yellow arrow). Note soft tissue emphysema (blue arrows), left pneumothorax (yellow circle) and left pleural effusion (orange circle).

The chest drainage was functioning, with active aspiration. On day two post-VATS, there was a clear worsening of subcutaneous emphysema (extension to the legs), with chest drainage under active low pressure aspiration. The patient underwent a chest CT scan that showed an enlarged bronchiolar structure in the anterior and lateral aspect of the left upper lobe, with 35 mm length, in communication with the pleural space through a 3 mm hole, consistent with a BPF. This fistula caused extensive superficial and deep soft tissue emphysema (in the entire length of the covered trunk), a large pneumomediastinum, and a moderate hydropneumothorax on the left (Figures 2-5).

**Figure 6:**
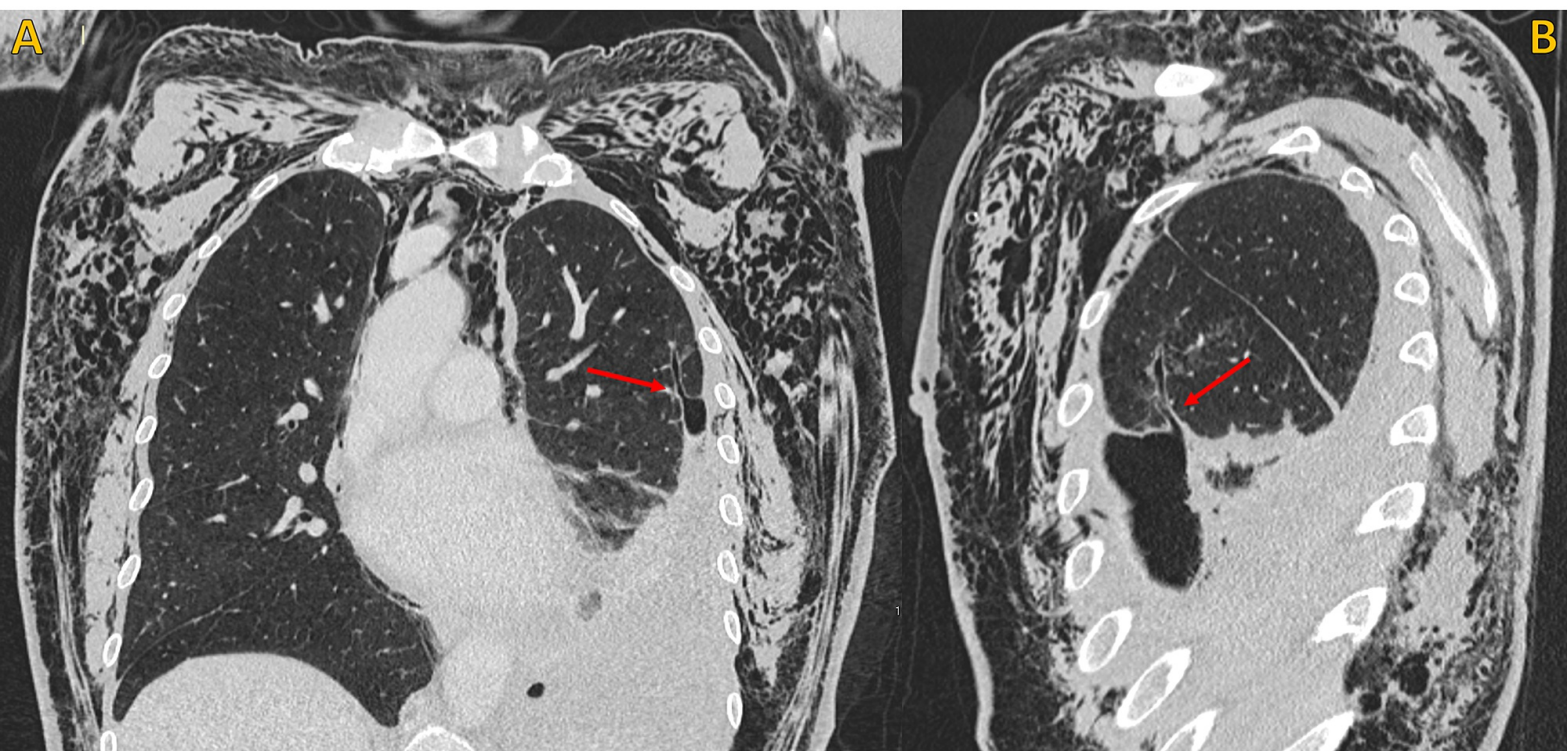
Non-contrast chest CT, lung window Non-contrast chest CT, lung window. A) Oblique coronal plane and B) Oblique sagittal plane demonstrates persistence of the bronchopleural fistula, with similar air-leak point (red arrow).

**Figure 7:**
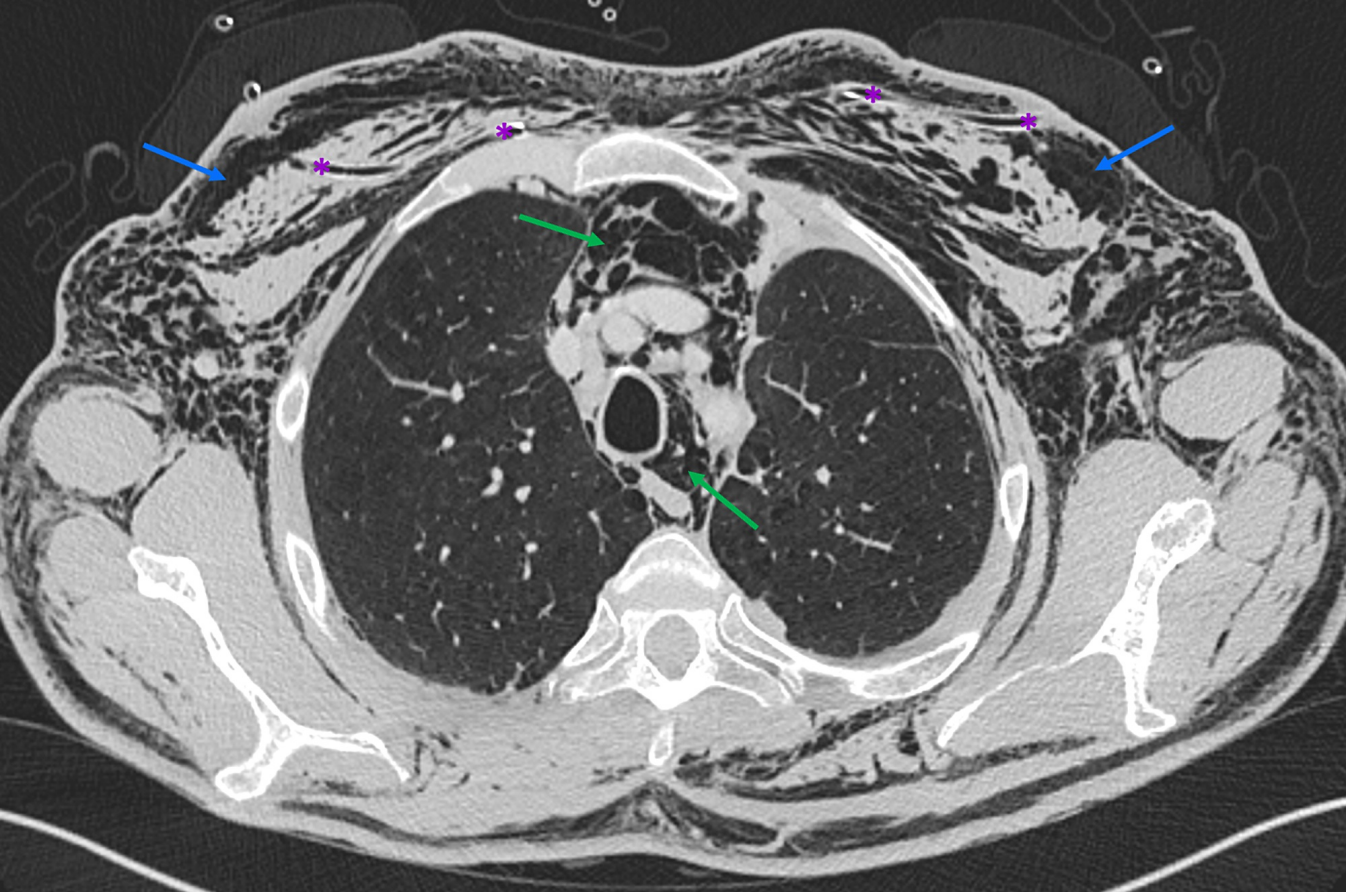
Non-contrast chest CT, lung window, and axial plane Non-contrast chest CT, lung window, and axial plane show slight reduction in the extent of pneumomediastinum (green arrows) and subcutaneous emphysema (blue arrows). Note bilateral subcutaneous tubes (purple asterisks).

**Figure 8:**
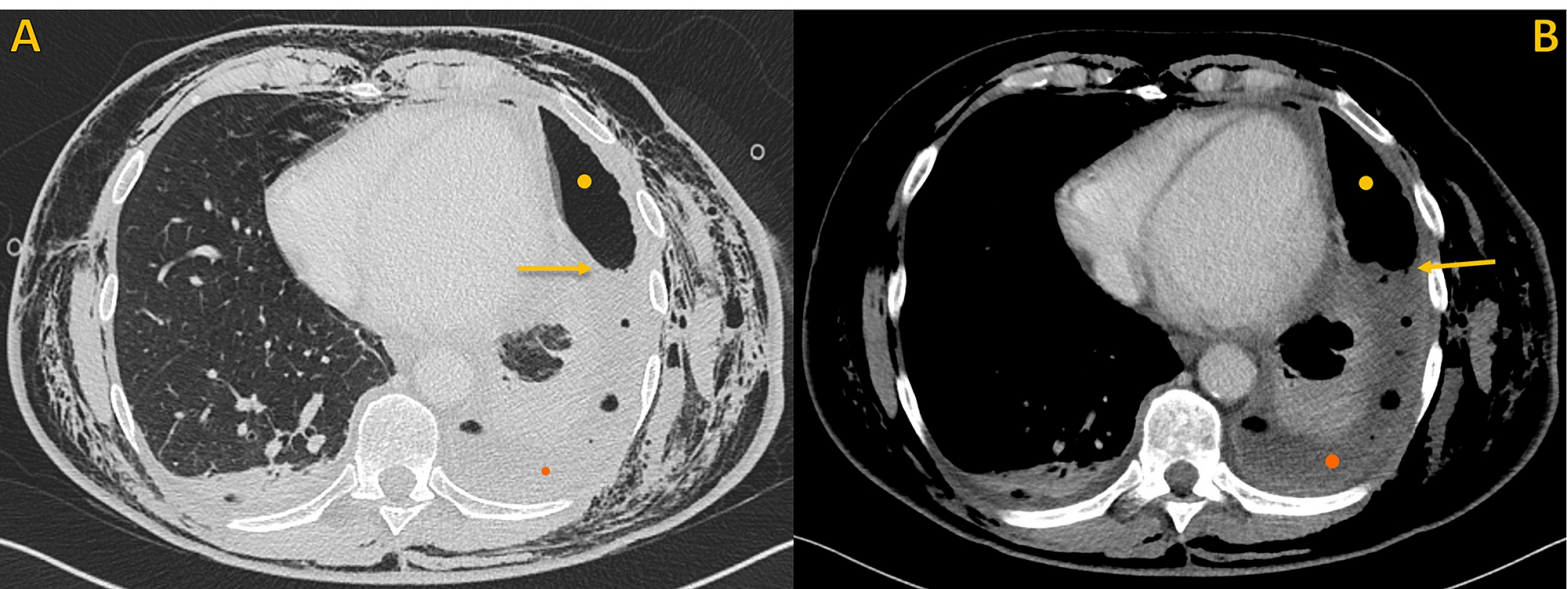
Non-contrast chest CT, same axial plane Non-contrast chest CT, axial images. A) Lung window and B) Soft tissue window, demonstrates reduction of the left pneumothorax (yellow circle) and the air-fluid level of the hydropneumothorax (yellow arrow). Note left pleural effusion (orange circle), with multiple air bubbles inside.

A multidisciplinary consensus was in favour of a conservative approach, consisting of maintaining a low pressure aspiration and surveillance. During hospitalization, the patient remained hemodynamically stable, without respiratory distress or fever. However, severe subcutaneous emphysema persisted, and hence, on day 10 post-VATS, two subcutaneous chest drains were placed in the anterior and upper chest wall, with a clear positive effect within the first hours. On day 12 post-VATS, the chest tube was accidentally externalized motivating a new chest CT scan, which showed persistence of the BPF, however with a slight improvement of the pneumomediastinum, subcutaneous emphysema, and left pneumothorax, despite a refilled left pleural effusion (nevertheless not to the volume seen at baseline) (Figures 6-8).

It was decided not to put another chest tube since the pneumothorax chamber had reduced and the patient remained asymptomatic. Subcutaneous chest drains were removed on day 15 post-VATS.

The anatomopathological study of pleural biopsies revealed chronic granulomatous inflammation, without evidence of neoplastic infiltration, which motivated another diagnostic thoracentesis on day 18 post-VATS. This time, the *M. tuberculosis* PCR test in the pleural fluid was positive. The patient was thus diagnosed with pleural tuberculosis and initiated antibacillary therapy.

**Figure 9:**
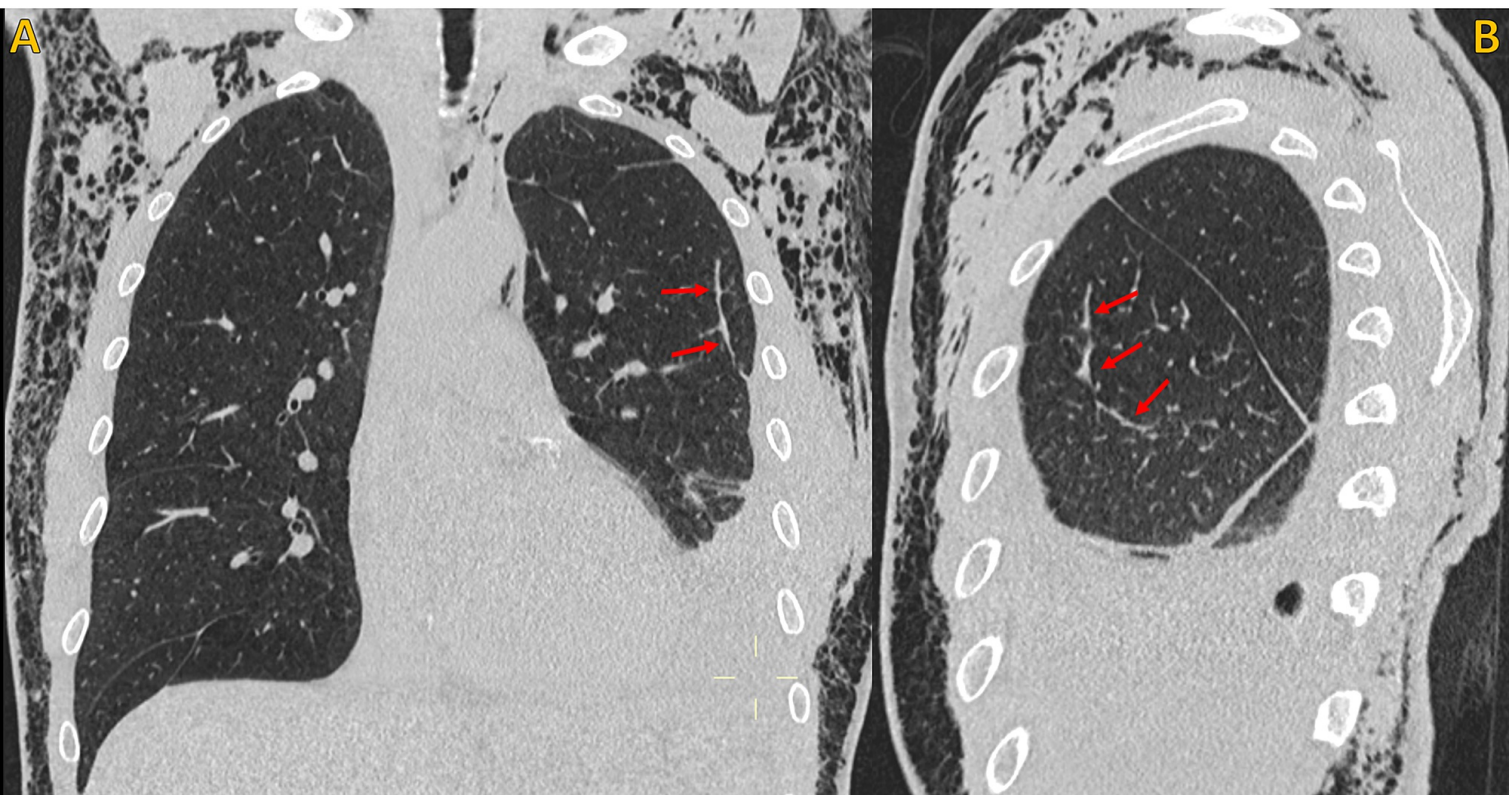
Non-contrast chest CT, lung window Non-contrast chest CT, lung window. A) Oblique coronal plane and B) Oblique sagittal plane showing that there is no longer evidence of the peripheral enlarged bronchiolar structure on the left lung neither the point of its communication with the pleural space. In its location, there is now an elongated and filiform structure, with soft tissue density and a fibrous and slightly retractable aspect, sequelae findings derived from the closure of the bronchopleural fistula (red arrows).

**Figure 10:**
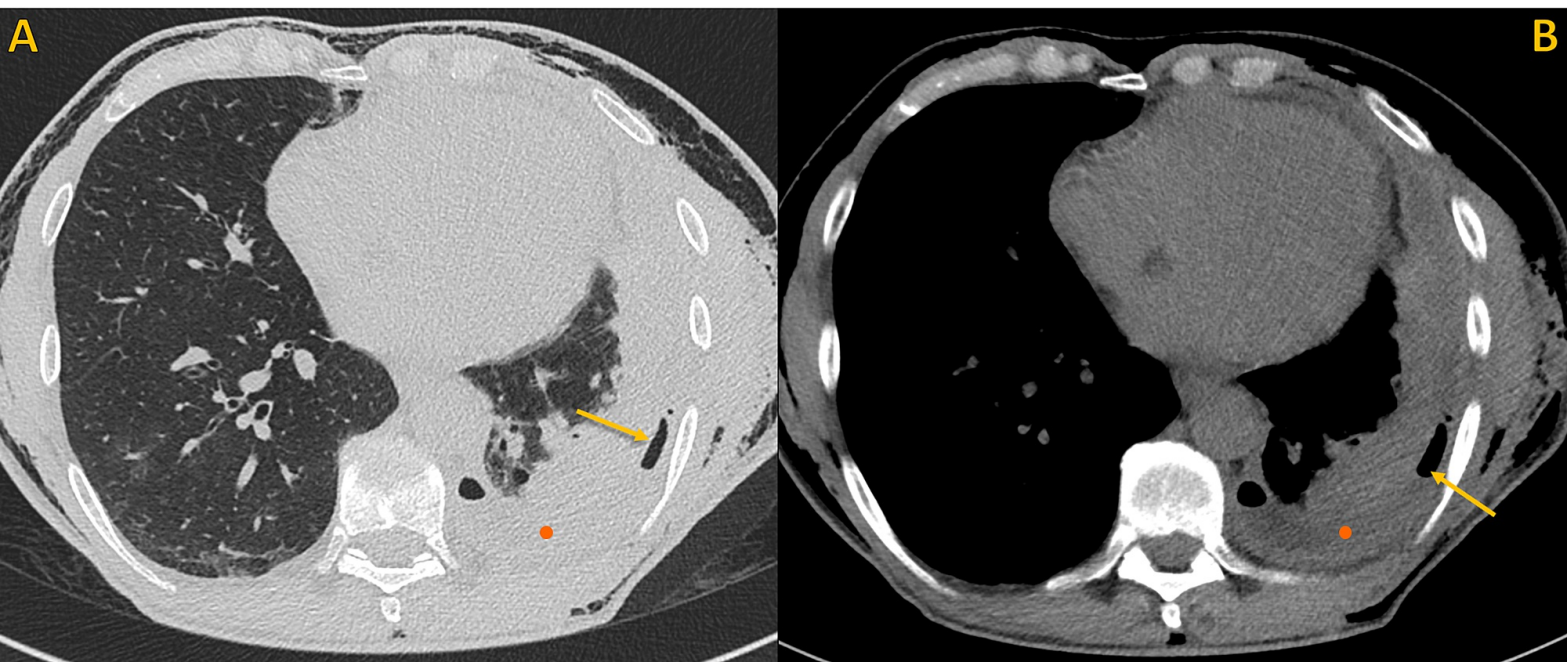
Non-contrast chest CT, same axial plane Non-contrast chest CT, same axial plane. A) Lung window and B) Soft tissue window, showing reexpansion of the left lung base, smaller left pleural effusion (orange circle), and a minimal pneumothorax (yellow arrow).

Follow-up chest imaging exams showed obliteration of the BPF, reexpansion of the left lung, reduced left pleural effusion, and a minimal ipsilateral pneumothorax, to be monitored in an outpatient setting (Figures 9-10).

**Figure 11:**
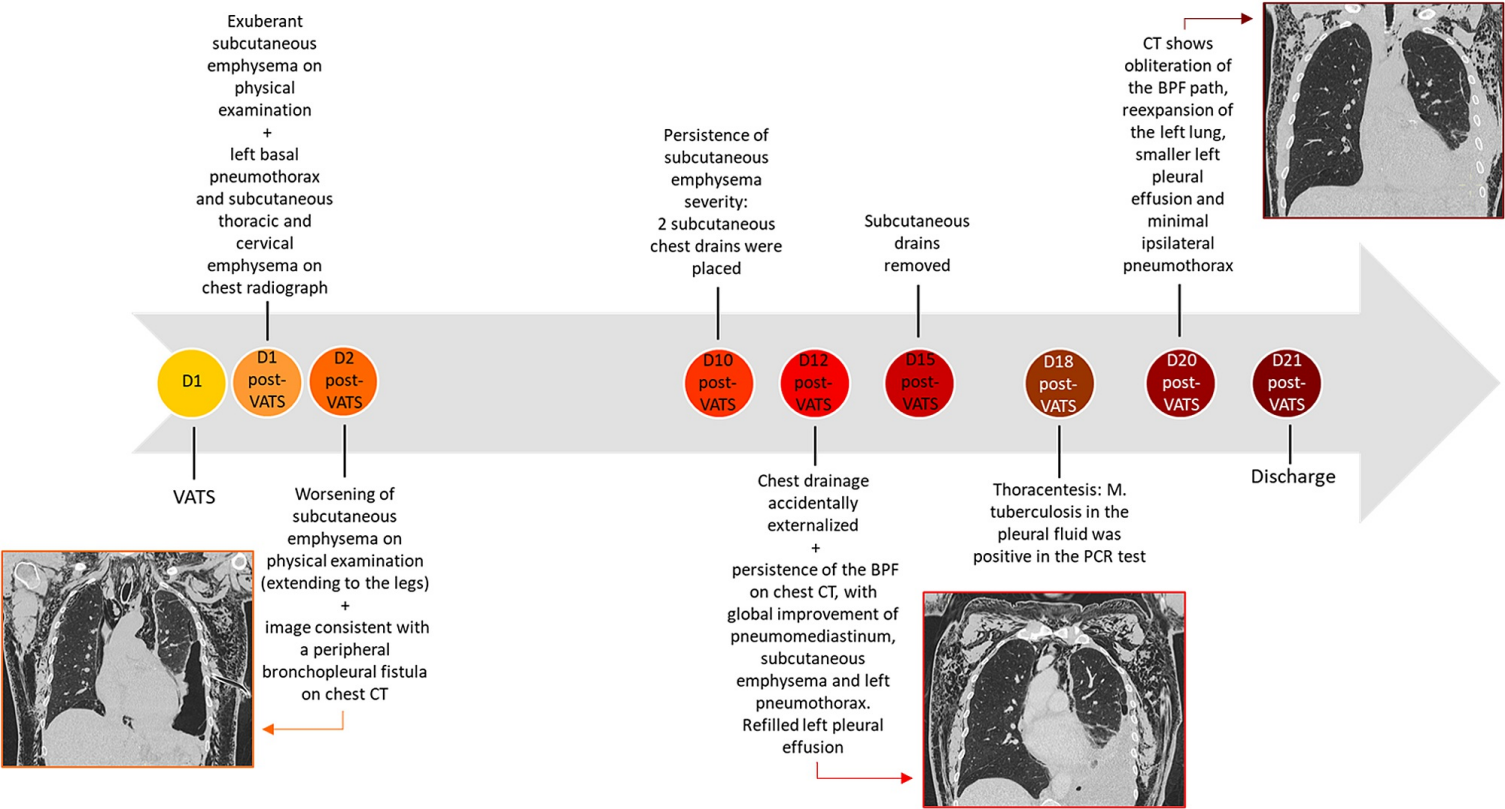
Diagram of the hospitalization timeline VATS: video-assisted thoracic surgery; BPF: bronchopleural fistula; PCR: polymerase chain reaction.

At discharge, the patient was clinically well. Mycobacteriological cultures were ongoing on Löwenstein-Jensen medium and BD BACTEC™ (Becton Dickinson, Sparks, MD, USA). The timeline of hospitalization is summarized in Figure 11.

## Discussion

A BPF is defined as a communication between the pleural space and the bronchial tree or the lung parenchyma, being a relatively rare pathology [1-4]. It presents most commonly as a postoperative complication of pulmonary resection [2,5]. Less common causes of BPFs include neoplasms (tumor extension into pleural space or tumor necrosis after chemo and/or radiotherapy), suppurative lung processes (infected pulmonary infarctions, necrotizing pyogenic pneumonia, tuberculosis, or septic pulmonary emboli), chronic obstructive pulmonary disease (COPD), traumatic chest injury (blunt and penetrating lung wounds or pneumatoceles) and other iatrogenic etiologies (complications of thoracentesis, chest tubes, pleural or lung biopsies and positive-pressure ventilation support) [1-2,4-6].

BPFs can be classified as central (communication between the main bronchus or the trachea and the pleural space) or peripheral (communication between the pleural space and a distal airway, either segmental bronchi of the lung parenchyma). Central BPFs are bigger, usually result from trauma or post-procedure complications, and require surgical repair [3-6]. Peripheral BPFs are smaller and typically caused by neoplasms, suppurative lung necrosis, bronchiectasis, or iatrogeny [3-6]. Iatrogenic BPFs, either central or peripheral, characteristically manifests within two weeks after the procedure. However, precocious or delayed manifestations may occur. When seen within the first four days after surgery, a BPF is usually related to dehiscence or technical issues, often requiring early reexploration and reclosure [2].

If acute, the typical clinical presentation includes dyspnea, cough, hypotension, and persistence of air leak (manifested by increased subcutaneous emphysema). Imaging studies may also show other signs of air leak persistence, besides subcutaneous emphysema, such as increasing pneumothorax, shifting of the mediastinum, or drop/disappearance of pleural effusion which becomes replaced by air (due to loss of the fluid through the tracheobronchial tree) [2,5]. Subacute and chronic presentations are commonly associated with infectious processes, having more atypical and insidious manifestations, such as weight loss, malaise, cough, and fever [2,5]. 

Unlike central BPFs, peripheral BPFs cannot be evaluated by bronchoscopy and are often suspected clinically. Therefore, imaging modalities play an essential role in the diagnosis of this entity [5-6]. CT remains the gold standard to diagnose, localize, and characterize (number and size) BPFs, also allowing treatment planning and surveillance [4-6]. However, the air-leak point on peripheral BPFs is rarely detected [3]. Imaging findings of BPFs include direct visualization of the communication between the bronchial tree or the lung parenchyma and the pleural cavity; increasing pneumothorax or a pneumothorax that does not resolve after placing a pleural drainage; the appearance of gas in a previous pleural effusion - hydropneumothorax; pneumomediastinum; progressive subcutaneous emphysema and contralateral mediastinal shift [1,4].

BPF may carry high morbidity and mortality rates, depending on the underlying cause, being associated with prolonged hospital stay [2,4]. Treatment options include conservative measures (medical treatment and pleural drainage), endoscopic treatment with glue, coils or sealants (in small peripheral BPFs, not bigger than 8 mm) and surgery (stent implantation or closing of the BPF, usually in central or big peripheral BPFs) [2,6]. Currently, there is no consensus regarding the best therapeutic approach, that should be wisely tailored to address each particular case. Treatment of peripheral BPF is controversial and remains challenging [5]. Imaging has a very important role in the therapeutic decision since some evidence suggests that if a peripheral BPF is visible on a CT it will usually need surgical treatment [3]. Yet, some more recent literature argues that conservative treatment may initially be sufficient, even if a peripheral BPF is evident on a CT scan [5]. Regardless, recurrence of pneumothorax and air-leak persistence are the main factors that influence the decision towards invasive procedures [3].

In our case, the patient developed extensive and marked subcutaneous emphysema very early, on the first day after an invasive thoracic procedure. Despite having no other symptoms, an iatrogenic BPF was suspected given the procedure-time related event. Consequently, the patient underwent a CT scan that confirmed this hypothesis. Although our patient presented two factors that would favour a more interventional therapeutic approach (early development of the BPF and its visualization on CT), the fact that the BPF was relatively small, peripheral, and occurred in a stable clinical asymptomatic setting, led to the choice of a conservative treatment. Indeed, invasive approaches are more consensually indicated for large fistulas (air-leak point greater than 3 mm) [2,3]. With time, clinical improvement (consisting of reduction of pneumothorax, pneumomediastinum, and subcutaneous emphysema) further substantiated the chosen treatment as being the most appropriate and effective. The pleural effusion did not significantly decrease during hospitalization since its cause (*M. tuberculosis*) was diagnosed later. Predictably, it will gradually disappear under the antibacillary regime.

## Conclusions

A BPF is a rare condition and may have a poor prognosis, depending on the underlying etiology. The most common cause is iatrogenic, specifically, post-pulmonary resection. A high index of clinical suspicion is essential for the diagnosis of BPF, with imaging playing an unparalleled role both in its diagnosis and management. CT scan remains the gold standard imaging modality in the diagnosis and characterization of BPFs that will impact therapeutic options. There is still no consensus regarding treatment, although some criteria have been proposed to standardize the decision for an invasive approach. This case demonstrates that conservative treatment can be effective, even when isolated CT criteria for invasive treatment are met, emphasizing the importance of a clinical-imaging integrated approach as the best way to avoid disconnected care.
